# Association between False Positive Misclassification and Modifiable Factors in Alzheimer's Disease Prediction Models among a Diverse Community Based Cohort

**DOI:** 10.1002/alz70856_098395

**Published:** 2025-12-24

**Authors:** Melissa Petersen, Fan Zhang, James Hall, Sid E. O'Bryant

**Affiliations:** ^1^ Institute for Translational Research, University of North Texas Health Science Center, Fort Worth, TX, USA; ^2^ University of North Texas Health Science Center, Fort Worth, TX, USA

## Abstract

**Background:**

Blood based biomarkers have been increasingly utilized and accepted as a means for determining diseases such as Alzheimer's disease (AD). Many of the tests are applied with relatively high accuracy rates, however, often these tests misclassify individuals as either having the disease but do not (false positive[FP]) or as not having the disease but do (false negative[FN]). This study sought to investigate potential underlying modifiable factors that could explain or contribute to the misclassification.

**Method:**

*n* = 1376 participants enrolled in the Health and Aging Brain Study‐Health Disparities were included for data analysis. Plasma AD biomarkers were generated utilizing Single Molecule Array technology and run on the HD‐X imager. Biomarkers included Amyloid Beta (Aβ) 42, 40, total tau, ptau181, and neurofilament light chain (NfL). Support Vector Machine models were run utilizing a combination of the plasma biomarkers to predict diagnostic status (AD or cognitively unimpaired). Those participants who were classified by the SVM models as a FP were included for follow‐up analyses utilizing Fisher's exact test. Modifiable risk factors of depression (determined by the Geriatric Depression Scale), worry (determined by the Penn State Worry Questionnaire), and hypothyroidism (determined by medical review) were included as predictors within the models. Significance was set at *p* <0.05.

**Result:**

Among Hispanics (*n* = 35/293 were FP), in the SVM model that utilized the Aβ42/40 ratio, there was a significant association between those who were classified as FP and hypothyroidism (*p* = 0.024) while a trend towards significance was found for Blacks (*n* = 139/729 were FP; *p* = 0.053) with no relationship found for non‐Hispanic Whites (NHW). Among non‐Hispanic whites (*n* = 82/354 were FP), in the SVM model that utilized Aβ 40, 42, the ratio and NfL, a significant association was found between those who were classified as FP and both depression (*p* = 0.023) and worry (*p* = 0.042). No association was found between the select modifiable factors and those classified as FN

**Conclusion:**

Differences were shown across race/ethnic group in the link between those classified as FP and modifiable factors with a medical modifier found to be associated for Blacks and Hispanics while psychiatric modifiers were found to be associated for NHWs. This highlights potential therapeutic avenues.

